# Qualitative study of barriers to cervical cancer screening among Nigerian women

**DOI:** 10.1136/bmjopen-2015-008533

**Published:** 2016-01-11

**Authors:** Fatima Isa Modibbo, Eileen Dareng, Patience Bamisaye, Elima Jedy-Agba, Ayodele Adewole, Lawal Oyeneyin, Olayinka Olaniyan, Clement Adebamowo

**Affiliations:** 1 Department of Microbiology and Parasitology, National Hospital Abuja, Abuja, Nigeria; 2 Department of Research, Institute of Human Virology, Abuja, Nigeria; 3 Department of Public Health and Primary Care, University of Cambridge, Cambridge, UK; 4 Department of Nursing Services, National Hospital, Abuja, Nigeria; 5 Department of Non-communicable Disease Epidemiology, London School of Hygiene and Tropical Medicine, London, UK; 6 Mother and Child Hospital, Ondo, Nigeria; 7 Department of Obstetrics and Gynecology, National Hospital, Abuja, Nigeria; 8 Department of Epidemiology and Public Health, University of Maryland School of Medicine, Baltimore, Maryland, USA; 9 Institute of Human Virology and Greenebaum Cancer Centre, University of Maryland School of Medicine, Baltimore, Maryland, USA

**Keywords:** PUBLIC HEALTH, QUALITATIVE RESEARCH

## Abstract

**Objectives:**

To explore the barriers to cervical cancer screening, focusing on religious and cultural factors, in order to inform group-specific interventions that may improve uptake of cervical cancer screening programmes.

**Design:**

We conducted four focus group discussions among Muslim and Christian women in Nigeria.

**Setting:**

Discussions were conducted in two hospitals, one in the South West and the other in the North Central region of Nigeria.

**Participants:**

27 Christian and 22 Muslim women over the age of 18, with no diagnosis of cancer.

**Results:**

Most participants in the focus group discussions had heard about cervical cancer except Muslim women in the South Western region who had never heard about cervical cancer. Participants believed that wizardry, multiple sexual partners and inserting herbs into the vagina cause cervical cancer. Only one participant knew about the human papillomavirus. Among the Christian women, the majority of respondents had heard about cervical cancer screening and believed that it could be used to prevent cervical cancer. Participants mentioned religious and cultural obligations of modesty, gender of healthcare providers, fear of disclosure of results, fear of nosocomial infections, lack of awareness, discrimination at hospitals, and need for spousal approval as barriers to uptake of screening. These barriers varied by religion across the geographical regions.

**Conclusions:**

Barriers to cervical cancer screening vary by religious affiliations. Interventions to increase cervical cancer awareness and screening uptake in multicultural and multireligious communities need to take into consideration the varying cultural and religious beliefs in order to design and implement effective cervical cancer screening intervention programmes.

**Strengths and limitations of this study**

- We identified several group-specific barriers to successful cervical cancer screening among Nigerian women and showed that religious and cultural factors have significant impact on health-seeking behaviours that should not be underestimated.
- Our findings have the potential to influence the implementation and uptake of cervical cancer screening in multicultural societies.
- Participants in our study were relatively well educated compared with the general population and may have provided responses that are not generalisable.
- We would need to further evaluate our findings from this qualitative study in a larger population-based survey.

## Introduction

Cervical cancer is the fourth most common cancer in women, with an estimated 528 000 new cases occurring globally in 2012.^1^ In Nigeria, it is the second most common female cancer, with an age-standardised incidence rate of 34.5 per 100 000 and incidence/mortality ratio of 0.6.^1–3^ The global burden of cervical cancer is unevenly distributed throughout the world, with developing countries accounting for over 80% of all new cases.^4^

The incidence of cervical cancer in developed countries has fallen significantly in recent decades due to the implementation of population-wide, cytology-based screening programmes.^4^ Similar programmes have either not been successful or are generally lacking in Sub-Saharan Africa and other low- and middle-income countries (LMICs). To overcome these difficulties, screening methods such as visual inspection techniques based on low-cost technologies have been developed and implemented in several LMICs with varying degrees of success.^5–7^ More recently, several high and middle-income countries have introduced screening based on human papillomavirus (HPV) DNA testing.^8^ ^9^

There are several barriers to the uptake of cervical cancer screening in LMICs; however, few studies have evaluated these barriers, particularly those related to cultural and religious differences in complex societies such as Nigeria. These barriers include low levels of knowledge of cervical cancer, limited awareness of prevention and early detection methods, fear of stigma associated with cancer diagnosis, concerns about spousal disapproval of screening, and concerns about violations of religious and cultural obligations of modesty during screening procedures.^4^ ^10^ ^11^

It is imperative that these barriers are reduced or eradicated and appropriate mechanisms are instituted to enhance the use of screening services.^11^ ^12^ In this study we explored the knowledge, attitude and beliefs of Nigerian women about cervical cancer prevention and focused on religious and cultural factors that may influence cervical cancer screening behaviour.

## Methods

### Study design and setting

**Figure 1:**
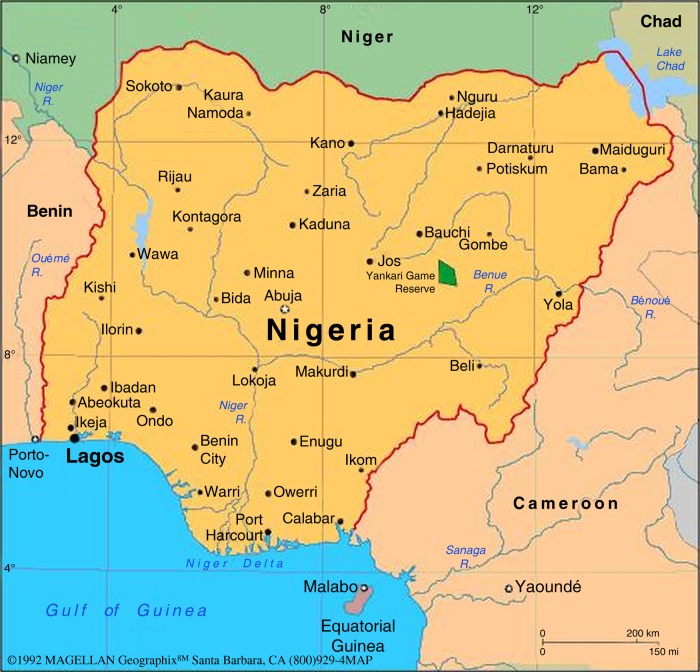
Map of Nigeria showing location of study sites; National Hospital Abuja in North Central Nigeria and Mother and Child Hospital, Ondo State, in South Western Nigeria.

We used focus group discussions (FGDs) so that we could elicit new information, free from researchers’ preconceived expectations in order to learn about group and cultural norms and behaviours associated with cervical cancer prevention among Nigerian women.^13^ This approach facilitates the flexible expression of ideas and experiences that might be left underdeveloped in a structured interview format, and its iterative data collection and research question format enables thorough exploration of participants’ perspectives, which quantitative methods do not allow. We conducted FGDs at two hospitals in Nigeria, where the Institute of Human Virology Nigeria (IHVN) works with site staff to provide cervical cancer screening services. These hospitals are the National Hospital, Abuja, Federal Capital Territory (FCT) located in North Central Nigeria and the Mother and Child Hospital, Ondo State located in the South Western region of the country (figure 1).

### Selection of participants and study sample

Participants were recruited through purposive sampling. Participants were eligible if they were (1) women resident in the North Central or South Western regions of the country, (2) age 18 or older, (3) not currently suffering from cervical cancer and (4) self-identified as Muslims or Christians. We did not exclude women who had family or close acquaintances with a present or past diagnosis of cervical cancer. We conducted in-person visits to churches, markets and the general outpatient clinics of participating hospitals to identify potential participants. Potential participants were screened by a brief in-person interview to assess eligibility, and eligible participants were provided venue and time details for the FGD. Six of 55 women (11%) who were approached to participate in the FGDs declined. All of these women cited time constraints as their reason for declining.

### Focus group discussions

We conducted four FGDs between August and October 2014, two at each of the hospitals where the study was implemented. In order to encourage free exchange of ideas, focus groups were conducted according to the faiths of the participants, one for Muslims and the other for Christians and we achieved a heterogeneous (by age and socioeconomic status) group with at least 10 participants in each FGD. All participants provided written informed consent.

The focus groups were conducted by FIM (a physician), EJ-A (a physician-epidemiologist) and PB (a nurse), all of whom, along with CA (physician-epidemiologist), have more than 4 years of experience in the design and conduct of qualitative research. FIM and EJ-A have had postgraduate training in qualitative research methodology at the West African Bioethics Training Program (PI—CA). EJ-A and CA have published papers using qualitative research methodology. All these researchers, except CA are women. AA, LO and OO are male gynaecologists who provide cervical cancer screening at the research sites and participated in the implementation of the research. None of the researchers had any relationship with the study participants before the start of the study.

All four FGDs were carried out in English language using pidgin or local terms when necessary and lasted approximately 60–90 min without interference or the presence of other people. At each FGD, using an FGD guide that we developed on the basis of previous literature and using predetermined domains relevant to the objectives of the study, one of the two researchers (FIM or EJ-A), assisted by PB, moderated the discussions. We pilot-tested the FGD guide among 15 women in Abuja identified through purposive sampling, before the start of the study. We informed participants that the reason for carrying out the research study was to determine barriers that might exist to cervical cancer screening, especially related to religious and cultural factors, in an attempt to ascertain ways of improving participation rates in cervical cancer screening programmes. We then addressed all questions they had, after which we took informed consent. We audiorecorded each FGD, and two independent members of the research team transcribed the recordings. Handwritten notes were also taken during the interviews and were used to supplement the audio recordings. The sample size was determined based on data saturation.^13^

### Data analysis

The transcribed data were analysed based on the content analysis technique using a combination of deductive and inductive processes with Atlas.ti V.7.5.2 software for qualitative analysis. Transcripts were not returned to the participants for any comments or corrections. Two of the authors, ED and EJ-A, created a priori codes based on our research objective, as a general framework, while allowing for emergent concepts from the discussions. For each code, a description of the code was written in the code manager to provide contextual information. Coding was an iterative process, with the first level of coding generating initial codes which were evaluated by ED and EJ-A for a second and third level of coding. Related codes were grouped together using prefixes related to the study objective (using shared characteristics), and codes were colour coded using the shared prefix titles for each specific objective. We grouped codes into families according to shared themes. ED and EJ-A created semantic networks representing hierarchical relationships between concepts after a review of the codes. We analysed each code to identify emerging opinions and themes for the research objective. We kept detailed reflective and thematic memos during the process of coding to provide contextual information that developed during the coding process. All the authors reviewed the codes at a data analysis review meeting and subsequently modified and agreed on a set of codes. We used the query tools and Boolean operators to interrogate the codes and obtain the results that we present in this paper. We used the consolidated criteria for reporting qualitative research (COREQ-32) in describing the results.^14^

### Quality assurance

We used the Word Cruncher and Word Cloud tools in Atlas.ti for content analysis to improve the quality of codes by identifying themes that may not have been previously coded. These tools determine the relative frequency of occurrence of all words in all four FGD transcripts. We did not identify any missed themes using this process.

### Ethical considerations

Ethics approval was obtained from the National Health Research Ethics Committee of Nigeria (NHREC/01/01/2007--01/08/2014).

## Results

### Participant description

A total of 49 women (25 from the South West and 24 from the North Central regions) participated in this study. The mean (SD) age of participants was 33 (9.9) years. Most of the participants (83.7%) had completed at least 12 years of formal education. The majority (63.3%) were married, and 59.0% were employed. About one-third of the participants (38.8%) had been screened for cervical cancer in the past. Table 1 provides details of the characteristics of the participants.

**Table 1:** Sociodemographic description of participants

	All participantsN=49	ChristianN=27	MuslimN=22
Site			
North Central (Abuja)	24 (49.0)	14 (51.9)	10 (45.5)
South West (Ondo)	25 (51.0)	13 (48.1)	12 (54.5)
Age, years (mean±SD)	33±9.9	36±9.4	30±9.6
Level of education			
Some primary school	1 (2.0)	0 (0.0)	1 (4.5)
Completed primary school	2 (4.1)	2 (7.4)	0 (0.0)
Some secondary school	5 (10.2)	3 (11.1)	2 (9.1)
Completed secondary school	15 (30.6)	9 (33.3)	6 (27.3)
Some University/post-secondary	7 (14.3)	3 (11.1)	4 (18.2)
Completed university/post-secondary	7 (14.3)	5 (18.6)	2 (9.1)
Some postgraduate	1 (2.0)	1 (3.7)	0 (0.0)
Completed postgraduate	11 (22.5)	4 (14.8)	7 (31.8)
Marital status			
Single	14 (28.6)	7 (25.9)	7 (31.8)
Married	31 (63.3)	17 (63.0)	14 (63.6)
Widowed	3 (6.1)	2 (7.4)	1 (4.6)
Separated	1 (2.0)	1 (3.7)	0 (0.0)
Occupation			
Civil servant	11 (22.4)	9 (33.3)	2 (9.1)
Professional	9 (18.4)	2 (7.4)	7 (31.8)
Skilled manual	9 (18.4)	3 (11.1)	6 (27.3)
Student	6 (12.2)	1 (3.7)	5 (22.7)
Unemployed	14 (28.6)	12 (44.5)	2 (9.1)
Frequency of attendance at religious meetings			
Daily	12 (24.5)	5 (18.5)	7 (31.8)
Weekly	34 (69.4)	22 (81.5)	12 (54.5)
Monthly	2 (4.1)	0 (0.0)	2 (9.1)
Never	1 (2.0)	0 (0.0)	1 (4.6)
Ever screened for cervical cancer	19 (38.8)	16 (59.3)	3 (13.6)

Except where indicated, all values are n (%).

### Awareness and knowledge about cervical cancer

#### Awareness of cervical cancer

Most participants had heard about cervical cancer (table 2). The most common means of hearing about it was through the mass media and at health talks from healthcare workers in hospitals. Interestingly, one participant recalled hearing about it during a talk given in a commuter bus. However, none of the Muslim participants in the FGD conducted in South Western Nigeria had ever heard about cervical cancer.

**Table 2:** Comparison of key findings on knowledge, attitudes and beliefs of participants

	Christian women		Muslim women	
	North Central (Abuja FGD)	South Western (Ondo FGD)	North Central (Abuja FGD)	South Western (Ondo FGD)
Awareness of cervical cancer	All of the women in this group had heard about cervical cancer	Most of the women in this group had heard about cervical cancer	Most of the participants in the Abuja Muslim women FGD had heard about cervical cancer	None of the women in this group had ever heard of cervical cancer
Causes of cervical cancer	Poor knowledge of the cause of cervical cancer; one of the participants in this group had the misconception that cervical cancer could result from wizardry	Poor knowledge of the cause of cervical cancer. Participants in this group expressed beliefs about the use of charms and cervical cancer being inflicted on women by men	Displayed poor knowledge of the cause of cervical cancer. Less belief in the use of charms than the Christian women	Displayed poor knowledge of the cause of cervical cancer
Symptoms of cervical cancer	Poor knowledge of the symptoms of cervical cancer	Poor knowledge of the symptoms of cervical cancer	Poor knowledge of the symptoms of cervical cancer	No previous knowledge of the symptoms of cervical cancer
Awareness of human papillomavirus	Only one participant in this group had ever heard of the human papillomavirus	No previous knowledge of the human papillomavirus	No knowledge of the human papillomavirus	No previous knowledge of the human papillomavirus
Treatment options for cervical cancer	Christian FGD participants in Abuja did not think that there was any effective traditional treatment for cervical cancer. They were of the opinion that it could be treated if diagnosed early by screening (see and treat), chemotherapy, surgery and ‘spirituality’	In the Christian women FGD in Ondo, participants noted that cervical cancer could be treated if diagnosed early. They did not believe that traditional medicine could be used to treat cervical cancer	Most Muslim FGD participants in Abuja believed that cervical cancer can be treated and few participants claimed awareness of traditional treatment modalities for it	Participants in the Ondo Muslim women FGD had poor knowledge of treatment options for cervical cancer
Cervical cancer screening	Participants in this group were more aware of cervical cancer screening and more willing to engage with the healthcare system. More than half of the participants in this group had been screened. Participants were not particular about being screened by a female or male healthcare provider	Participants in this group had heard about cervical cancer screening and were more likely to accept screening. Participants were also indifferent to being screened by either a male or female healthcare provider	Participants among the Abuja Muslim women had heard about cervical cancer screening, though none had been screened. Reasons for reluctance to be screened included the need for spousal support and permission before screening, need for a female doctor or female chaperone to be present during screening procedure	None of the participants in this group was aware of cervical cancer screening
*Attitudes and beliefs*				
Perception of personal risk	Most expressed the belief that cervical cancer is becoming a significant problem in Nigeria and that anyone could be at risk of cervical cancer	Most of the women in this group believed that cervical cancer is becoming more common in Nigeria and they could be at risk of cervical cancer	Most of the Abuja Muslim women believed that cervical cancer is becoming a significant problem in Nigeria. They expressed belief that they could be at risk of cervical cancer	None of the participants in this group previously believed that cervical cancer is becoming a significant problem or that they could get cervical cancer
Barriers to cervical cancer screening	No barriers to cervical cancer screening were reported in this group. While some opined that they would prefer a female provider, many were happy to be screened by a male provider if a female chaperone was present	Participants in this group identified lack of awareness as a major barrier to cervical cancer screening	The Abuja Muslim women highlighted the need for husband's permission, modesty concerns, lack of awareness, cultural discrimination and discomfort as barriers to cervical cancer screening	Participants in this group identified a strong preference for a female healthcare provider before they would accept being screened
Acceptability of self- sampling	Expressed preference for sample collection in the hospital by a healthcare provider rather than self-sampling	Expressed preference for sample collection in the hospital by a healthcare provider	Expressed preference for sampling by a female healthcare provider rather than self-sampling	Expressed preference for sampling by a female healthcare provider rather than self-sampling. One participant in this group was of the opinion that healthcare providers are more capable of taking the samples correctly

FGD, focus group discussion.

Among participants who had heard about cervical cancer, most did not understand the part of the body affected by it or where the cervix is located. A few participants identified cancer of the cervix by describing the cervix as the mouth of the womb or the birth canal. Several participants believed it was the same condition as cancer of the breast, while a few others believed it was cancer of the uterus. Some other discussants thought that it was cancer of the hips. During the FGDs, we found that, when some participants volunteered the names ‘cancer of the mouth of the womb’ or ‘cancer of the birth canal’, several other participants in the FGDs, who had heard of cervical cancer but did not know what part of the body was affected, were able to identify the location of the cervix.

#### Causes of cervical cancer

Discussants had some misconceptions about the cause of cervical cancer (table 2). These varied by religion and geographical location. Among the Christian women in the Abuja FGDs, one of the most common misconceptions was that cervical cancer could result from wizardry.

I heard that…On that talk show somebody say is…They use to send it, maybe it is a kind of charm, if a woman offend a man, the man now charm her that she will not have sex again. Then that place will now start to decay. That was what they said. Abuja Christian FGD participant

Among the Muslim women in the Abuja FGD, various vaginal health practices were thought to cause cervical cancer (table 2). These practices include the use of toilet paper or cloth as sanitary pads during menstrual periods and the insertion of herbs into the vagina.

…I think you know women have a lot of…like herbs that they insert into their vagina. So me I felt…you know sometimes you see tablets, sometimes it's these local herbs or something. The first time I learnt about it, from I think a friend was telling me. That like in Niger (a State in Northern Nigeria), you know they are used to buying these women herbs. That most of women there have cervical cancer. Abuja Muslim FGD Participant

Most respondents believed that having multiple sexual partners would increase the risk of having cervical cancer.

she was talking about sexual intercourse, having a multitude of partners even though in one partner but it is not safe for you because he may have other girl friends, Which he can contract from them too. Abuja Muslim FGD participant

In addition to the causes mentioned above, one of the Abuja Christian FGD participants also mentioned that it could be hereditary.

#### Symptoms of cervical cancer

Although most participants in the FGDs did not demonstrate a good understanding of the part of the body affected by cervical cancer, they were able to mention some of the symptoms of cervical cancer. From all FGDs, except the Ondo Muslim FGD, abnormal vaginal bleeding was recognised as a symptom of cervical cancer by the majority of the participants (table 2). Some respondents further characterised this to be bleeding during sexual intercourse, prolonged menstrual periods or abnormal menstrual cycles. Other symptoms that were mentioned included abnormal growth from the vagina, offensive vaginal discharge, abdominal pain and back pain.

I think they said irregular menses, or at times, heavy flow of menstrual blood. That is one. I think that is what I heard. Ondo Christian FGD participant

Offensive discharge, bleeding during sexual intercourse. Abuja Christian FGD participant.

#### Cervical cancer screening

There was limited knowledge of the use of screening as a means of preventing cervical cancer (table 2). This differed by religion but not by geographic location. Among the Christian women, the majority of respondents had heard about cervical cancer screening and believed that it could be used to prevent cervical cancer. Respondents who did not believe that screening could be used for prevention of cervical cancer felt that it could only be used for early detection of cervical cancer.

I think if you go early enough and you do your screening you will know when if you have it at all you will know early, and from there you start the treatment. Which is easier than you knowing it when it's late. Abuja Christian FGD participant

While many of the Muslim women in Ondo had not heard of cervical cancer screening, Muslim women in Abuja had heard about screening for cervical cancer; however, they had limited knowledge of its use for prevention. Some of the Muslim FGD participants in Ondo said that the only screening they had heard of was for breast cancer.

Among the participants who knew about cervical cancer screening, the majority believed that it is only required for premenopausal women who are sexually active and that the screening interval should be yearly.

#### Cervical cancer treatment options

Regardless of location, the Christian women identified treatment options for cervical cancer to include early detection, screening, chemotherapy, ‘spirituality,’ ‘exercise.’ One participant noted that preventive hysterectomy was also an option saying

Another way is that if a woman have delivered the children she wanted, I think the womb can be removed. Abuja Christian FGD participant

Participants in Abuja did not feel that there was any effective traditional treatment for cervical cancer, although one participant noted that traditional treatment was available for breast cancer but not for cervical cancer saying

I have seen on breast cancer but not heard of cervical cancer. Abuja Christian FGD participant

Most Muslim FGD participants in Abuja believed that cervical cancer can be treated, and a few participants claimed awareness of traditional treatment modalities for it. Participants who were aware of traditional treatment modalities for cervical cancer provided further insight by noting that, when women had symptoms suggestive of cervical cancer, they started traditional treatment before visiting a healthcare facility. In these instances, such participants noted that they could not tell much about the efficacy of the traditional treatment, as they were not certain if what was being treated was indeed cervical cancer. In the Christian FGD in Ondo, participants noted that cervical cancer could be treated if diagnosed early.

Yes it can be treated if it has not damaged the womb. If it is still around the cervix and it has not gone very far, it can be treated. Ondo Christian FGD participant

These participants did not believe that traditional medicine could be used to treat cervical cancer.

#### Barriers to cervical cancer screening

All participants, except the Muslim women in Ondo, opined that cervical cancer was becoming a significant problem in Nigeria, as they hear about the disease more commonly now than in the past. One respondent described rural–urban disparities in the awareness of cervical cancer saying

This awareness of cervical cancer is limited to the city… Ondo Christian FGD participant

All participants, except those in the Muslim FGD in Ondo, believed that they could develop cervical cancer as long as they were engaged in sexual relationships and with increasing age. One participant said

it is all about having sex and many multiple sexual partners. Abuja Christian FGD participant

Although most participants believed that they have some personal risk of developing cervical cancer, several expressed unwillingness to be screened. Some of the barriers to screening that respondents described included modesty concerns, gender of healthcare providers, fear of status disclosure, fear of contracting other illnesses in the hospitals, discomfort during the screening process, lack of awareness of screening programmes, denial of disease condition, discrimination, and requiring husband’s permission before screening. These barriers varied by religion across the geographical regions.

All FGD participants identified cultural norms of modesty as a barrier to seeking cervical cancer screening; however, the participants in the Muslim FGDs were more emphatic about this barrier, citing an additional religious basis for their concern. A participant put it this way

Islamically it is not good to expose yourself outside, except to your husband. Ondo Muslim FGD participant

A participant noted that there can be exceptions to this norm in cases of illness and explained how Islam teaches the practice of preventive measures.

Even in our religion, our mentor in Islam, Muhammadu Rasurulillahi has even told us—When you are in community and you can perceive a kind of a sickness that is being spread among the people or even in the forest that you should leave that place and go to area where you will not contact it. Ondo Muslim FGD participant

The participants in the Muslim FGD in both geographical regions expressed a strong preference for a female healthcare provider.

Normally….Muslim women do not like to open themselves to the other… man. I prefer female doctors to attend to the Muslim woman. To me, I prefer to go female hospital, because they will see our private parts. I don't really, really like it at all at all. Ondo Muslim FGD participant.

Some participants further explained that they had failed to go through screening in the past when they realised the service was to be provided by a male healthcare provider. Participants in the FGD for Muslim women in Ondo claimed that Muslim women face some discrimination at healthcare facilities because their mode of dressing readily identifies their faith.

The way they are treating us in the hospital, as if we are not part of the community. Even the female doctors in the hospital, before they even attend to you, they will already be abusing you from head to toe as if you don't know anything, as if you are not part of the community. If you want us to go for screening in the hospital, these women that are coming, at least they should be kind to them and be patient with them. Ondo Muslim FGD participant

Among all the Christian FGD participants, while there was a preference for female healthcare providers, they did not mind being attended to by a male healthcare provider. All participants opined that they would be more comfortable if there was a female chaperone present in the examination room.

Some respondents expressed concerns about confidentiality of results.

Some women will not want any other person to know their status. You know if you have infection, that tendency of thinking that so they know I am carrying this thing…. Ondo Christian FGD participant

Participants expressed a lack of confidence in the healthcare system and opined that they could be infected with other diseases either from the screening equipment or from other sources or procedures within the healthcare facility.

I may be thinking maybe, I will contact another disease. Like this Ebola we have been hearing this time around now. Most of the doctor that have been treating the patients, they are the ones catching the disease. If I go for screening now, maybe something may have happen, I don't know. That is the part of the reason that have been scaring us from going. Ondo Muslim FGD participant

Most participants said that they required their husband’s permission before being screened. This view was more prevalent among the Muslim respondents. Some felt they needed to let their husbands know before attending screening clinics because of financial reasons, stating that the husband would need to provide the funds. One participant noted that it would be important to inform her husband because this would make disclosure and discussion of abnormal results, if found during screening, easier. Other reasons proffered for requiring their husband’s permission were religious obligation and trust in their spouse’s opinion.

Most participants were not aware that there were screening options that allow the participant to take the cervical samples by themselves. However, when they considered this option, the majority expressed a preference for a healthcare provider to take the samples in a healthcare facility as opposed to self-sampling. The main reason for this preference was the belief that they would be unable to take adequate samples of good quality even with instructions.

Because the reason is that when you give us the thing to screen at home we won't have something to open it (private part) very well. And we may not do it properly. Ondo Muslim FGD participant.

#### Practices

The majority of the FGD participants (61.2%) had never been screened for cervical cancer. Participants attributed this low level of screening uptake to modesty concerns, lack of awareness of screening, lack of husband's permission to be screened, fear of a positive result, and belief that it is better to be ignorant of the disease than to go in search of it. However, several participants who had not been screened for cervical cancer were willing to be screened if a free screening test was provided. They also offered suggestions on how cervical cancer screening could reach a wider range of women by the use of mobile screening clinics, dissemination of information in churches and mosques, and use of text messages via mobile phones.

## Discussion

Our study shows that awareness of cervical cancer was not uniform among different groups of women in our population. When compared with the Christian FGD participants, the Muslim women were less likely to be aware of cervical cancer especially in the Ondo South Western FGD, where none of the participants were aware of cervical cancer. This finding may be because there have been more public health campaigns in churches and at Christian gatherings. It may also be a reflection of the scope of social and ancillary programmes that accompany the practices of Christianity compared with Islam in our study communities. We also found a higher prevalence of reluctance to engage with the healthcare system among Muslim participants. There is a need to provide educational campaigns that are specifically designed to reach Muslim women, as recommended by others.^15^ The most effective way of conducting such campaigns may require specific engagement with Muslim women's organisations and close liaison with Muslim religious and opinion leaders because Muslim women may not attend mosques as frequently as men. The differences in cervical cancer awareness between the Muslim FGD participants based on geographic regions were apparent and this may be due to differences in sociocultural dynamics regardless of religious beliefs between the two groups.

Most of the women in our study were aware of cervical cancer, but only a third had ever been screened and only one participant had ever heard of the association between HPV and cervical cancer. This is similar to findings from other low-resource settings, which found that the knowledge of women about cervical cancer is ‘elementary’ at best and that there was a high prevalence of ignorance about causes, symptoms and treatment options for cervical cancer.^16^ Other studies in Nigeria have found only moderate levels of awareness of cervical cancer even among female healthcare and professional workers.^17^ ^18^ The lack of knowledge about cervical cancer may be related to the hidden anatomical location of the cervix in contrast with the breast, which is located on the surface and is familiar to most of our participants.

Some of the women in our study knew cancer of the cervix as ‘cancer of the mouth of the womb’ or ‘cancer of the birth canal’. To be successful, public health messages to promote cervical cancer screening should build on names and terminologies that are already in use in the community. Our study participants stated that their main sources of information on cervical cancer were the mass media and hospital health talks. This is similar to the findings from other studies carried out in Nigeria and Ethiopia.^18^ ^19^ Given the low level of knowledge about methods of cervical cancer prevention in this study, additional modalities for dissemination of health information including use of text messages and campaigns in public spaces including mass transport vehicles should be actively explored.

Several participants recognised the role of a sexually transmitted infection in cervical cancer, but only one participant mentioned an association with HPV. With increasing use of HPV DNA testing as primary modality for cervical cancer screening in high- and middle-income countries and the role of HPV vaccination in prevention of cervical cancer, there is a need to improve awareness of the association of HPV with cervical cancer in order to enhance the uptake of these modern prevention and screening interventions in this population.^8^ ^9^ ^20^ There was a higher prevalence of beliefs about an association between practices such as the insertion of herbs into the vagina and cervical cancer among Muslim women and this may be related to prevailing vaginal and sexual health practices within those communities. While previously under-reported in Nigeria, such practices may alter the vaginal microenvironment and facilitate the acquisition and persistence of HPV infection.^21^ Further studies are needed to obtain a good understanding of the materials that are used in such practices and the reasons for their use. Women who do not engage in these practices may falsely believe that they are not at risk of cervical cancer and fail to appreciate that the most important risk factor is an active sexual life.

Beliefs that cervical cancer may be caused by charms deployed by men who are unhappy with their female sexual partners were more prevalent among Christians in this study. These beliefs may reflect the growth of evangelical Christianity with its emphasis on the spiritual nature of illnesses in recent decades in Nigeria.^22^ They may also play to deep anxieties about sex and gender roles in these communities.^23^ Beliefs that cervical cancer is a sexually transmitted disease or may be associated with having multiple sexual partners were also prevalent among study participants. These beliefs may contribute to reluctance of women to participate in screening programmes because they would not want to be considered sexually promiscuous, harbouring a sexually transmitted disease, or having unhappy male sexual partners. The public health message about HPV infection and cervical cancer needs to be carefully crafted so that women learn that every sexually active woman is at risk from this very common viral infection and that there are no effective religious/spiritual remedies.

There was a prevailing notion among several participants that cervical cancer screening is only used for detection of cancer that is already present and the cancer may have a poor outcome even if treated. This limited knowledge of cervical cancer treatment, and its options, coupled with fear of receiving a diagnosis of cervical cancer during screening was a strong deterrent to accessing cervical cancer screening services. Other studies have found that the lack of awareness of cervical cancer and its causes, symptoms and treatment among women in Sub-Saharan Africa decreases their cervical cancer screening participation rates and increases their risk of developing cancer.^24^ ^25^ Educational programmes in support of cervical cancer screening programmes need to emphasise the value of the methods for primary prevention.

Although the majority of participants in the FGDs recognised their personal risk of cervical cancer, several expressed an unwillingness to be screened because of cultural and religious norms of modesty. This was expressed in several forms such as the lack of desire to disrobe for the pelvic examination necessary for cervical cancer screening in the clinic and concerns at being attended to by male healthcare providers. These concerns were more pronounced among the Muslim participants, and they related it to the teachings of their faith. Our finding on the preference for a female doctor among the Muslim women in our sample is similar to reports from a predominantly Muslim population in Kuwait where 78% of respondents expressed their preference for examination by a female doctor.^26^ The Islamic culture and religion has a specific tradition and lifestyle that shapes women's actions, behaviours, health practices, beliefs, expectations, gender roles, and self-care.^27^ These practices may influence a woman's decision not to have a physical examination or other healthcare interventions if the health provider is a male regardless of the severity of the health conditions.^28^ All participants agreed that, if it was absolutely necessary for them to be attended to by a male healthcare provider, then it would be helpful to have a female chaperone present.

Most participants indicated that they would need spousal financial and emotional support before attending screening services. In Africa, men have an important role to play in improving uptake of screening programmes and health interventions, so health education programmes for women's health need to engage spouses in order to increase their chances of success.^29^ ^30^ This spousal support is particularly important among Muslim women partly because of higher prevalence of spousal dependency because women are allowed only limited roles outside the home and because of injunctions of the Islamic faith. Some studies have found that women would not go for screening because they lacked their husband's support to access such services.^31^ A study in Tanzania found that, when husbands approved of screening, the likelihood of participating in screening increased significantly.^10^ Many of our study participants identified financial limitations as another factor that may affect their willingness to attend cervical cancer screening services. Several participants said that they would agree to participate in a screening programme if the screening test provided was free. The financial cost of screening was also one of the reasons for requiring spousal permission because the participants said they would need money from their spouses. A screening programme is therefore more likely to be successful if it is provided at no or low cost to participants.

An unexpected finding in our study was the perception of discrimination at healthcare facilities by Ondo FGD Muslim women who suggested that this prevents them from seeking care. This is an important finding especially since these same women were unaware of cervical cancer. Participants opined that their mode of dressing, which involves the wearing of a veil and long loose black gowns (the hijab), triggers a discriminatory response from healthcare providers. The Muslim women mentioned that they already feel that they are being discriminated against when they attend services such as the antenatal clinic. This therefore discourages them from returning to hospitals for other, particularly non-critical, services. Healthcare providers need to remain ethical and treat all patients equally. Perceived racial, ethnic, religious or cultural discrimination may alter health behaviour including cancer screening, leading to group-specific cancer health disparities.^32^ Health workers must be trained to avoid perceived or actual bias against any of the constituent communities they serve.

Participants in our study also showed evidence of low levels of trust in the healthcare system. There was concern about disclosure of status based on the screening result and fear of contracting other diseases while in the hospital environment. While there have been several studies of disclosure of status and its impact in the context of HIV/AIDS,^33^ there have been fewer studies of fear of disclosure as a barrier to cancer screening programmes in Africa where the disease remains highly stigmatised.^33^ ^34^ Patients in LMICs often harbour anxieties about the quality of services and the environment of hospitals. But this concern may have been heightened in our study participants because we conducted this study around the peak of public awareness of Ebola virus disease, where a high proportion of healthcare workers were affected by the disease.

We explored the attitude of our FGD participants to potential use of self-sampling for cervical cancer screening in Nigeria in this study. Participants expressed concerns about being able to take adequate samples and would rather have the samples taken by a trained provider in a healthcare facility. Similar concerns have been found in other studies and highlight the need to ensure that women are adequately trained before introduction of self-sampling techniques for cervical cancer screening.^35^ ^36^

Limitations of our study include the possibility that the FGD environment may have caused some participants to give answers they perceive to be more ‘socially acceptable’, while more reticent participants may have shied away from participation. There may also have been some selection bias in recruiting participants for the FGDs. Participants in our study were relatively well educated and they may have provided responses that are not generalisable to the general population. Another limitation to our study is the sample size; although we conducted two FGDs in each geographical region, we acknowledge that our findings of distinct experiences and perceptions among Muslim women in Ondo require further exploration. It is our plan to explore this further in a population-based survey.

## Conclusions

Our study adds valuable information to the knowledge, attitudes and practices of cervical cancer screening in Nigeria. We identified several barriers to successful cervical cancer screening among Nigerian women, some of which varied by religion. We showed that religious and cultural factors could have significant impact on health-seeking behaviours that should not be underestimated, and that there were significant differences in attitudes to cervical cancer screening among the two major religious groups in Nigeria. Our next steps are to further evaluate our findings from this qualitative study in a larger population survey.

## References

[1] Ferlay J, Soerjomataram I, Ervik M GLOBOCAN 2012 v1.0, Cancer Incidence and Mortality Worldwide: IARC Cancer Base No. 11. Lyon, France: International Agency for Research on Cancer, 2013 (cited 20 December 2013). http://globocan.iarc.fr

[2] Parkin DM, Ferlay J, Hamdi-Cherif M Cancer in Africa: epidemiology and prevention. Lyon: IARC Press, 2003.

[3] Jedy-Agba E, Curado MP, Ogunbiyi O Cancer incidence in Nigeria: a report from population-based cancer registries. Cancer Epidemiol 2012;36:e271–8. 10.1016/j.canep.2012.04.007 22621842 PMC3438369

[4] Ferlay J, Shin HR, Bray F Estimates of worldwide burden of cancer in 2008: GLOBOCAN 2008. Int J Cancer 2010;127:2893–917. 10.1002/ijc.25516 21351269

[5] Sankaranarayanan R, Budukh AM, Rajkumar R Effective screening programmes for cervical cancer in low- and middle-income developing countries. Bull World Health Organ 2001;79:954–62. 11693978 PMC2566667

[6] Blumenthal PD, Gaffikin L, Deganus S Cervical cancer prevention: safety, acceptability, and feasibility of a single-visit approach in Accra, Ghana. Am J Obstet Gynecol 2007;196:407.e1–8; discussion e8–9 10.1016/j.ajog.2006.12.031 17403438

[7] Mwanahamuntu MH, Sahasrabuddhe VV, Pfaendler KS Implementation of ‘see-and-treat’ cervical cancer prevention services linked to HIV care in Zambia. AIDS 2009;23:N1–5. 10.1097/QAD.0b013e3283236e11 19279439 PMC2747794

[8] Huh WK, Ault KA, Chelmow D Use of primary high-risk human papillomavirus testing for cervical cancer screening: interim clinical guidance. Gynecol Oncol 2015;136:178–82. 10.1016/j.ygyno.2014.12.022 25579107

[9] Lazcano-Ponce E, Lőrincz AT, Torres L Specimen self-collection and HPV DNA screening in a pilot study of 100,242 women. Int J Cancer 2014;135:109–16. 10.1002/ijc.28639 24615258

[10] Lyimo FS, Beran TN Demographic, knowledge, attitudinal, and accessibility factors associated with uptake of cervical cancer screening among women in a rural district of Tanzania: three public policy implications. BMC Public Health 2012;12:22 10.1186/1471-2458-12-22 22233530 PMC3299640

[11] Azaiza F, Cohen M Between traditional and modern perceptions of breast and cervical cancer screenings: a qualitative study of Arab women in Israel. Psychooncology 2008;17:34–41. 10.1002/pon.1180 17352007

[12] Guimond ME, Salman K Modesty matters: cultural sensitivity and cervical cancer prevention in muslim women in the United States. Nurs Womens Health 2013;17:210–17. 10.1111/1751-486X.12034 23773193

[13] Morgan DL Focus Groups as Qualitative Research 1997 (cited 15 May 2015). In: SAGE Research Methods (cited 15 May 2015);[32–46]. http://www.uk.sagepub.com/gray3e/study/chapter18/Book%20chapters/Planning_and_designing_focus_groups.pdf

[14] Tong A, Sainsbury P, Craig J Consolidated criteria for reporting qualitative research (COREQ): a 32-item checklist for interviews and focus groups. Int J Qual Health Care 2007;19:349–57. 10.1093/intqhc/mzm042 17872937

[15] Hasnain M, Menon U, Ferrans CE Breast cancer screening practices among first-generation immigrant muslim women. J Womens Health (Larchmt) 2014;23:602–12. 10.1089/jwh.2013.4569 24865517 PMC4089017

[16] Ali-Risasi C, Mulumba P, Verdonck K Knowledge, attitude and practice about cancer of the uterine cervix among women living in Kinshasa, the Democratic Republic of Congo. BMC Womens Health 2014;14:30 10.1186/1472-6874-14-30 24548698 PMC3937079

[17] Ayinde OA, Omigbodun AO Knowledge, attitude and practices related to prevention of cancer of the cervix among female health workers in Ibadan. J Obstet Gynaecol 2003;23:59–62. 10.1080/0144361021000043272 12623487

[18] Hyacinth HI, Adekeye OA, Ibeh JN Cervical cancer and pap smear awareness and utilization of pap smear test among Federal civil servants in North Central Nigeria. PLoS ONE 2012;7:e46583 10.1371/journal.pone.0046583 23049708 PMC3462186

[19] Getahun F, Mazengia F, Abuhay M Comprehensive knowledge about cervical cancer is low among women in Northwest Ethiopia. BMC Cancer 2013;13:2 10.1186/1471-2407-13-2 23282173 PMC3559275

[20] Makwe CC, Anorlu RI, Odeyemi KA Human papillomavirus (HPV) infection and vaccines: knowledge, attitude and perception among female students at the University of Lagos, Lagos, Nigeria. J Epidemiol Glob Health 2012;2:199–206. 10.1016/j.jegh.2012.11.001 23856501 PMC7320323

[21] Dareng EO, Ma B, Famooto AO Prevalent high-risk HPV infection and vaginal microbiota in Nigerian women. Epidemiol Infect. 2016;144:123–37. 26062721 10.1017/S0950268815000965 PMC4659743

[22] Ezeome ER, Anarado AN Use of complementary and alternative medicine by cancer patients at the University of Nigeria Teaching Hospital, Enugu, Nigeria. BMC Complement Altern Med 2007;7:28 10.1186/1472-6882-7-28 17850665 PMC2034592

[23] Heslop J, Banda R Moving beyond the “male perpetrator, female victim” discourse in addressing sex and relationships for HIV prevention: peer research in Eastern Zambia. Reprod Health Matters 2013;21:225–33. 10.1016/S0968-8080(13)41697-X 23684205

[24] Mupepi SC, Sampselle CM, Johnson TR Knowledge, attitudes, and demographic factors influencing cervical cancer screening behavior of Zimbabwean women. J Womens Health (Larchmt) 2011;20:943–52. 10.1089/jwh.2010.2062 21671779

[25] Francis SA, Battle-Fisher M, Liverpool J A qualitative analysis of South African women's knowledge, attitudes, and beliefs about HPV and cervical cancer prevention, vaccine awareness and acceptance, and maternal-child communication about sexual health. Vaccine 2011;29:8760–5. 10.1016/j.vaccine.2011.07.116 21855591

[26] Al Sairafi M, Mohamed FA Knowledge, attitudes, and practice related to cervical cancer screening among Kuwaiti women. Med Princ Pract 2009;18:35–42. 10.1159/000163044 19060489

[27] Salman KF Health beliefs and practices related to cancer screening among Arab Muslim women in an urban community. Health Care Women Int 2012;33:45–74. 10.1080/07399332.2011.610536 22150266

[28] Hammoud MM, White CB, Fetters MD Opening cultural doors: providing culturally sensitive healthcare to Arab American and American Muslim patients. Am J Obstet Gynecol 2005;193:1307–11. 10.1016/j.ajog.2005.06.065 16202719

[29] Hossain M, Mani KK, Sidik SM Knowledge and awareness about STDs among women in Bangladesh. BMC Public Health 2014;14:775 10.1186/1471-2458-14-775 25081860 PMC4246425

[30] Ezeonwu M Policy strategies to improve maternal health services delivery and outcomes in Anambra State, Nigeria. Health Care Women Int 2014;35:828–44. 10.1080/07399332.2014.925454 24911182

[31] Sawadogo B, Gitta SN, Rutebemberwa E Knowledge and beliefs on cervical cancer and practices on cervical cancer screening among women aged 20 to 50 years in Ouagadougou, Burkina Faso, 2012: a cross-sectional study. Pan Afr Med J 2014;18:175 10.11604/pamj.2014.18.175.3866 25419302 PMC4236918

[32] Crawley LM, Ahn DK, Winkleby MA Perceived medical discrimination and cancer screening behaviors of racial and ethnic minority adults. Cancer Epidemiol Biomarkers Prev 2008;17:1937–44. 10.1158/1055-9965.EPI-08-0005 18687583 PMC2526181

[33] Gourlay A, Birdthistle I, Mburu G Barriers and facilitating factors to the uptake of antiretroviral drugs for prevention of mother-to-child transmission of HIV in sub-Saharan Africa: a systematic review. J Int AIDS Soc 2013;16:18588 10.7448/IAS.16.1.18588 23870277 PMC3717402

[34] Dey S Preventing breast cancer in LMICs via screening and/or early detection: the real and the surreal. World J Clin Oncol 2014;5:509–19. 10.5306/wjco.v5.i3.509 25114864 PMC4127620

[35] Bansil P, Wittet S, Lim JL Acceptability of self-collection sampling for HPV-DNA testing in low-resource settings: a mixed methods approach. BMC Public Health 2014;14:596 10.1186/1471-2458-14-596 24927941 PMC4061776

[36] van Baars R, Bosgraaf RP, ter Harmsel BW Dry storage and transport of a cervicovaginal self-sample by use of the Evalyn Brush, providing reliable human papillomavirus detection combined with comfort for women. J Clin Microbiol 2012;50:3937–43. 10.1128/JCM.01506-12 23015677 PMC3503012

